# A Cyclic Nucleotide-Gated Channel, HvCNGC2-3, Is Activated by the Co-Presence of Na^+^ and K^+^ and Permeable to Na^+^ and K^+^ Non-Selectively

**DOI:** 10.3390/plants7030061

**Published:** 2018-07-26

**Authors:** Izumi C. Mori, Yuichi Nobukiyo, Yoshiki Nakahara, Mineo Shibasaka, Takuya Furuichi, Maki Katsuhara

**Affiliations:** 1 Institute of Plant Science and Resources, Okayama University, 2-20-1 Chuo, Kurashiki 710-0046, Japan; imori@okayama-u.ac.jp (I.C.M.); nobino8455@gmail.com (Y.N.); ps8c7lfe@s.okayama-u.ac.jp (Y.N.); smine@okayama-u.ac.jp (M.S.); furuichi@nagoya-ku.ac.jp (T.F.); 2 Department of Human Life Science, Nagoya University of Economics, 61-1 Uchikubo, Inuyama 484-8504, Japan

**Keywords:** cyclic nucleotide-gated channel, barley, sodium, potassium, roots

## Abstract

Cyclic nucleotide-gated channels (CNGCs) have been postulated to contribute significantly in plant development and stress resistance. However, their electrophysiological properties remain poorly understood. Here, we characterized barley CNGC2-3 (HvCNGC2-3) by the two-electrode voltage-clamp technique in the *Xenopus laevis* oocyte heterologous expression system. Current was not observed in *X. laevis* oocytes injected with *HvCNGC2-3* complementary RNA (cRNA) in a bathing solution containing either Na^+^ or K^+^ solely, even in the presence of 8-bromoadenosine 3′,5′-cyclic monophosphate (8Br-cAMP) or 8-bromoguanosine 3′,5′-cyclic monophosphate (8Br-cGMP). A weakly voltage-dependent slow hyperpolarization-activated ion current was observed in the co-presence of Na^+^ and K^+^ in the bathing solution and in the presence of 10 µM 8Br-cAMP, but not 8Br-cGMP. Permeability ratios of HvCNGC2-3 to K^+^, Na^+^ and Cl^−^ were determined as 1:0.63:0.03 according to reversal-potential analyses. Amino-acid replacement of the unique ion-selective motif of HvCNGC2-3, AQGL, with the canonical motif, GQGL, resulted in the abolition of the current. This study reports a unique two-ion-dependent activation characteristic of the barley CNGC, HvCNGC2-3.

## 1. Introduction

Sodium uptake into plant cells is suggested to be mediated by voltage-independent non-selective cation channels [1,2]. However, the molecular entity of the ion channel has not been identified. Cyclic nucleotide-gated channels (CNGCs) are argued to be one of the ion channels involved in Na^+^ influx [3].

CNGCs are ion channels that are activated by the binding of adenosine 3′,5′-cyclic monophosphate (cAMP) and/or guanosine 3′,5′-cyclic monophosphate (cGMP). In mammals, CNGCs are involved in the signal transduction of olfactory and visual sensing in a such way that environmental stimuli induce the elevation of cyclic nucleotide levels in the cell and in turn activate CNGCs leading to firing of neurons [4,5,6,7]. The activation of Ca^2+^-permeable CNGCs by an external stimulus results in the rise of intracellular Ca^2+^ concentrations, which in turn induce the excitation of sensory neurons [8].

Plant genomes possess a gene family of CNGC sequences [9,10,11,12,13], postulating diverse functions of CNGCs in plants. A number of CNGC genes have been predicted in plant genomes (for instance, 20 in Arabidopsis, 15 in rice and 9 in barley), whereas a handful of the CNGCs have been characterized electrophysiologically. Previous electrophysiological studies showed that the *Arabidopis thaliana* CNGC, AtCNGC2, enable K^+^ permeation when examined in the *Xenopus laevis* oocyte heterologous expression system [14,15]. Similarly, a transient expression of AtCNGC10 in human embryonic kidney cells promoted K^+^ currents [16]. Ion permeability of plant CNGCs was also investigated with indirect analyses by means of functional complementation of the K^+^-uptake-deficient mutants of *Saccharomyces cerevisiae* and *Escherichia coli* [17]. In addition to K^+^, several studied have reported that CNGCs facilitate Ca^2+^, using heterologous expression systems [18,19,20,21]. However, reports on electrophysiological properties of plant CNGCs are lacking apart from these studies. A few electrophysiological studies in vivo demonstrated activation of Ca^2+^ currents across plasma membranes by an application of membrane-permeable cAMP analogues in root, mesophyll and guard cells, although the corresponding *CNGC* genes were unidentified [22,23]. It was reported that voltage-independent cation (VIC) currents were inhibited with cAMP and cGMP in *Arabidopsis* root cell protoplasts, as opposed to initiating activation of current [24]. Recently, loss-of-function mutants *atcngc5* and *atcngc6* were shown to lack cGMP-activated Ca^2+^-permeable currents in guard and root cells, suggesting their function as Ca^2+^ permeable channels, although the electrophysiological properties of the channels were not characterized in detail [25]. 

Despite the dearth of knowledge of electrophysiological properties, genetic and molecular biological evidence suggesting the physiological significance of plant CNGCs has accumulated (for review, see [26]). The loss-of-function mutation in *AtCNGC16* reduced growth and viability of pollen, as well as seed yields [27]. The rice *OsCNGC13* is involved in fertility through function in the pistil [28]. The *Arabidopsis* mutant, *defense*, *no death1* (*dnd1*), was isolated based on the failure of hypersensitive response to avirulent pathogens. The causative gene, *DND1,* encoded *AtCNGC2* [29]. The knockout mutant of *AtCNGC1* demonstrated an attenuated gravitropism response in roots [30]. The *Arabidopsis* mutant, *constitutive expressor of PR gene22* (*cpr22*), which displayed a constitutive activation of a defense-response phenotype, possessed a large deletion spanning neighboring CNGC genes *AtCNGC11* and *AtCNGC12* [31,32]. Disruption of *AtCNGC10* conferred salt tolerance in *Arabidopsis* [33]. Heterologous over-expression of *Nicotiana tabacum CBP4* in *Arabidopsis* conferred heavy-metal tolerance [34,35]. Loss-of-function mutation of *CNGCb* of the moss, *Physconitrella patens*, and its ortholog in *Arabidopsis*, *AtCNGC2*, caused a heat-hypersensitive phenotype [36]. 

Plant genomes possess a family of *CNGC* genes (20 genes in the *Arabidopsis* genome, for instance), which are classified into four groups (group I, II, III and IV) based on similarity of the primary structure [12,13,37]. *AtCNGC10*, *AtCNGC11* and *AtCNGC12*, belonging to the group I, and *AtCNGC2* and *AtCNGC4*, belonging to the group IV, are involved in salt tolerance, defense response and thermal sensing [29,32,36,38,39]. *HvCNGC4* involving necrotic phenotype and constitutive expression of pathogenesis-related gene 1 of barley is also a member of the group IV [40]. *AtCNGC16* and *AtCNGC18*, which are involved in pollen growth, belong to group III [41,42,43]. It was shown that the *AtCNGC7* and *AtCNGC8*, the *Arabidopsis* group II CNGCs expressed in pollen tubes, were involved in male fertility [27]. The structural diversity among the groups implies the differentiation of biochemical and electrophysiological roles of each group. Relative to the other groups, the physiological significance and electrophysiological properties of group II have been poorly characterized.

In this study, we characterized the electrophysiological properties of the group-II CNGC of barley, HvCNGC2-3, using two-electrode voltage-clamp measurements in the heterologous expression system of *X. laevis* oocytes.

## 2. Results

### 2.1. Identification and Isolation of Group-II CNGC Genes from Barley Roots

**Figure 1:**
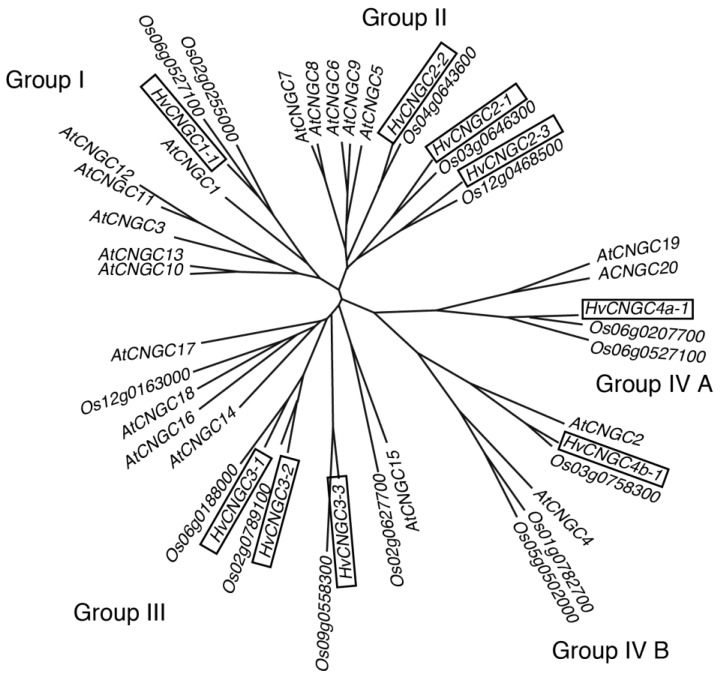
Phylogenetic tree of *CNGC* genes in plants. Barley *CNGC* genes are highlighted by boxes. Accession numbers of barley genes are described in Table S1. Arabidopsis Genome Initiative codes are available in Table S2. Rice Annotation Project identifiers are shown for rice CNGCs.

We identified nine nucleotide sequences of *CNGC* cDNA in barley from the public domain database, Barley DB (URL: http://www.shigen.nig.ac.jp/barley/), by a BLAST search against the barley full-length cDNA and expressed sequence tag (EST). Deduced amino-acid sequences of CNGCs of *Arabidopsis thaliana* (TAIR, URL: http://www.arabidopsis.org) and *Oryza sativa* (Rice Genome Annotation Project, URL: http://rice.plantbiology.msu.edu/index.shtml) were used as the query sequences. Phylogenetic analysis showed that nine identified cDNA sequences of *CNGC* genes were classified into four predicted groups (Figure 1). Accession numbers of these *HvCNGC* sequences are listed in Table S1.

**Figure 2:**
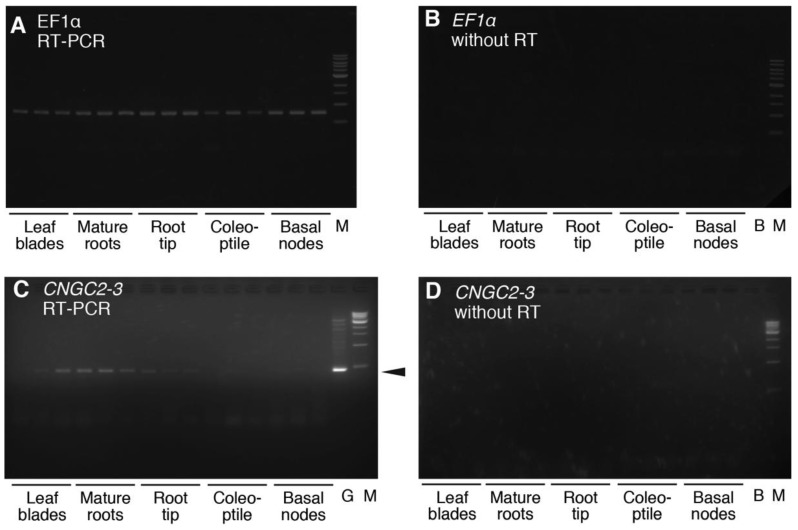
Expression analysis of *HvCNGC2-3* mRNA by reverse transcription-polymerase chain reaction (RT-PCR). DNA fragments of *HvEF1α* (**A**) and *HvCNGC2-3* (**C**) amplified with RT-PCR from three independently prepared cDNA samples were analyzed by agarose gel electrophoresis. G, *HvCNGC2-3* band amplified from genomic DNA of barley cultivar Haruna-Nijyo for comparison. Arrowhead indicates the position of *HvCNGC2-3*. M, DNA size marker ‘Without RT’ indicates PCR using the template without reverse-transcription reaction controls serving as a control experiment proving free of genomic DNA contamination in the samples using *HvEF1α* primers (**B**) and *HvCNGC2-3* primers (**D**).

We attempted to amplify DNA fragments of cDNA coding for three *CNGC* genes belonging to group II, *HvCNGC2-1*, *HvCNGC2-2* and *HvCNGC2-3* by reverse transcription-polymerase chain reaction (RT-PCR). RT-PCR analysis of *HvCNGC2s* mRNA and the internal standard, *HvEF1α*, was carried out from five different parts of the seedlings, these being leaf blades, mature roots, root tips, coleoptiles and basal nodes (Figure 2). Since the *EF1α* gene was used as the internal control of RT-PCR in rice [44], we designed primers to amplify barley *EF1α* (*HvEF1α*) (sequence of the primers are described in Material and Methods). Amplification of a single band of *HvEF1α* was nearly evenly detected in all five tissues (Figure 2A), while no bands were detected in controls without RT reaction (Figure 2B), indicating that *HvEF1α* was a suitable internal control for RT-PCR analysis of barley plantlets and the contamination of genomic DNA is negligible in the sample. An amplified DNA band that showed the same size with the genome-PCR control of *HvCNGC2-3* was detected in mature roots (Figure 2C). A weaker band was observed in root tips and leaf blades, and a very faint band was observed in basal nodes. No band was visible in the control without RT reaction (Figure 2D), indicating the RT-PCR band of *HvCNGC2-3* was not derived from contamination of genomic DNA. Full-length cDNA of *HvCNGC2-3* was then isolated from cDNA of roots as described in Methods and utilized for subsequent electrophysiological analyses. 

### 2.2. HvCNGC2-3 Is Activated by the Co-Presence of Na^+^ and K^+^, and Allows Permeation of Na^+^ and K^+^ Non-Selectively

**Figure 3:**
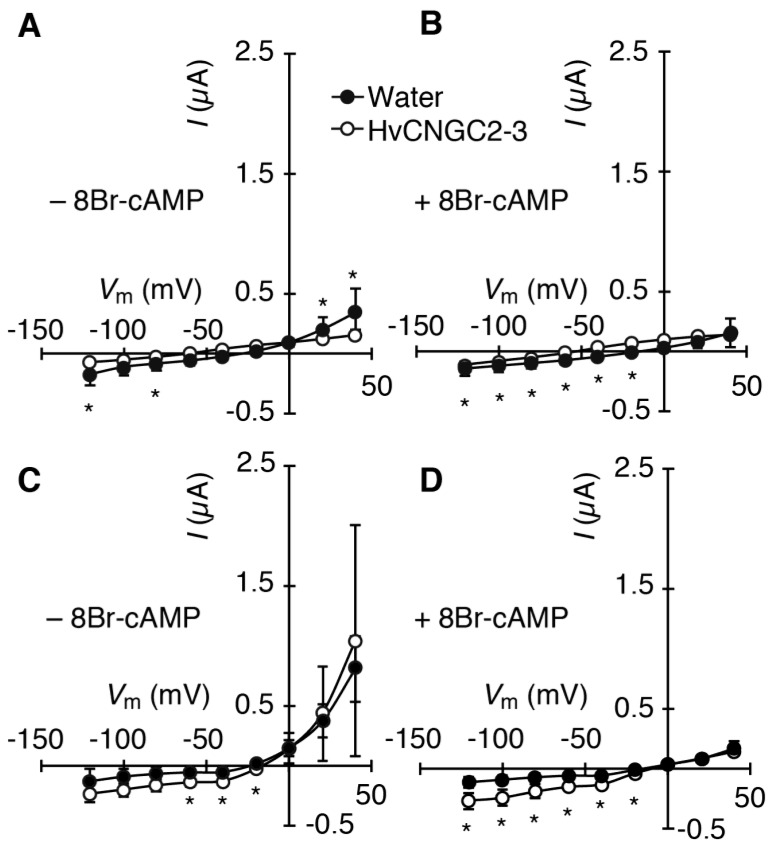
Current/voltage relationship of HvCNGC2-3-expressing oocytes in Na^+^ and K^+^ media. Currents were recorded by the two-electrode voltage-clamp technique. *HvCNGC2-3* cRNA-injected oocytes (open circles), water-injected control oocytes (closed circles). (**A**,**B**) bath solution containing a Na^+^ medium (96 mM NaCl); (**C**,**D**) bath solution containing a K^+^ medium (96 mM KCl). Oocytes were treated with (**B**,**D**) or without (**A**,**C**) 8Br-cAMP (10 μM) 30 min before measurement. Current values are means (*n* = 14 [panel A], 7 [panel B], 13 [panel C] and 7 [panel D] for water-injected control, and *n* = 6 [A], 6 [B], 13 [C] and 7 [D] for HvCNGC2-3) ± SD. *I*, membrane current. *V*m, membrane potential. Asterisks indicate significant difference of the means between *HvCNGC2-3* cRNA-injected oocytes and water-injected control oocytes (Student’s *t*-test, *p* < 0.05).

Ion currents in *X. laevis* oocytes were examined with TEVC in a Na^+^ medium containing 96 mM NaCl (Figure 3A,B). The oocytes, which were injected with water or *HvCNGC2-3* cRNA, exhibited essentially no current both in the absence (−0.11 ± 0.07 µA at −100 mV) and presence (−0.12 ± 0.05 µA at −100 mV) of 8Br-cAMP (Figure 3A,B, respectively). In a K^+^ medium containing 96 mM KCl, an outward-rectifying current was observed (Figure 3C), which was suppressed by the addition of 8Br-cAMP in the bath solution (Figure 3D). No significant difference in currents was found between the water-injected and the cRNA-injected oocytes in the tested conditions. This suggests that the outward currents did not correspond to HvCNGC2-3 but rather endogenous currents of the oocytes. The treatment with 8Br-cGMP showed essentially the same result as 8Br-cAMP in the Na^+^ and K^+^ media (Figure S1).

**Figure 4:**
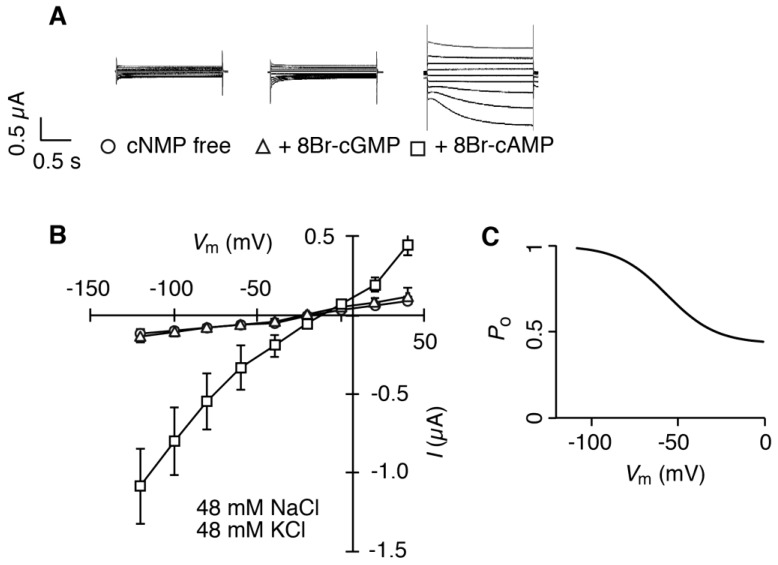
Current/voltage relationship of *HvCNGC2-3*-expressing oocytes in a 1:1 mixture of Na^+^ and K^+^ media. Currents were recorded by the two-electrode voltage-clamp technique in a bathing solution containing 48 mM NaCl and 48 mM KCl in the absence of a pre-incubation with cyclic nucleoside monophosphates (cNMP-free, circles), and in the presence of a pre-incubation with 10 µM 8Br-cGMP (triangles) or 8Br-cAMP (squares) for 30 min. (**A**) representative raw recording traces; (**B**) current/voltage curve. Error bars indicate standard deviation (*n* = 7, 13 and 9 for cNMP-free, 8Br-cGMP and 8Br-cAMP, respectively). *I*, membrane current. *V*m, membrane potential; (**C**) *P*_o_, apparent open probability fitted with modified Boltzmann equation.

Typical readouts of the current from *HvCNGC2-3* cRNA-injected oocytes in a bathing solution containing a 1:1 mixture of Na^+^ and K^+^ media are shown in Figure 4A. In the presence of 8Br-cAMP, a weakly voltage-dependent slow-activated current was observed (Figure 4A,B). Time constant of activation (τ) was determined as 0.59 ± 0.24 s (mean ± standard deviation, *n* = 9). In contrast to 8Br-cAMP, a negligible current was detected in the presence of 8Br-cGMP or in the absence of cNMPs (Figure 4). A noticeable current was only observed in the co-presence of Na^+^ and K^+^ in the bathing solution, and not in the presence of either ion individually (compare Figure 4 with Figure 3). Importantly, no current was observed in water-injected oocytes in the presence of both Na^+^ and K^+^ in the bathing solution (−0.06 ± 0.01 µA at −100 mV) (Figure S2). The relative open probability (*P*_o_) of HvCNGC2-3 tended to be higher at more negative potentials in the media containing 1:1 Na^+^ and K^+^ (apparent gating charge and half activation membrane potential were determined as 0.016 [95% confidence interval was 0.010–0.021] and −62 mV [95% confidence limits were −69 and −56 mV], respectively) (Figure 4C). Minimum *P*_o_ was estimated as 0.43. Outward currents were also observed, which exhibit slow inactivation kinetics (Figure 4A). Other alkali cations failed to substitute for Na^+^ or K^+^ to activate 8Br-cAMP-activated currents in *HvCNGC2-3* cRNA-injected oocytes (Figure S3).

**Figure 5:**
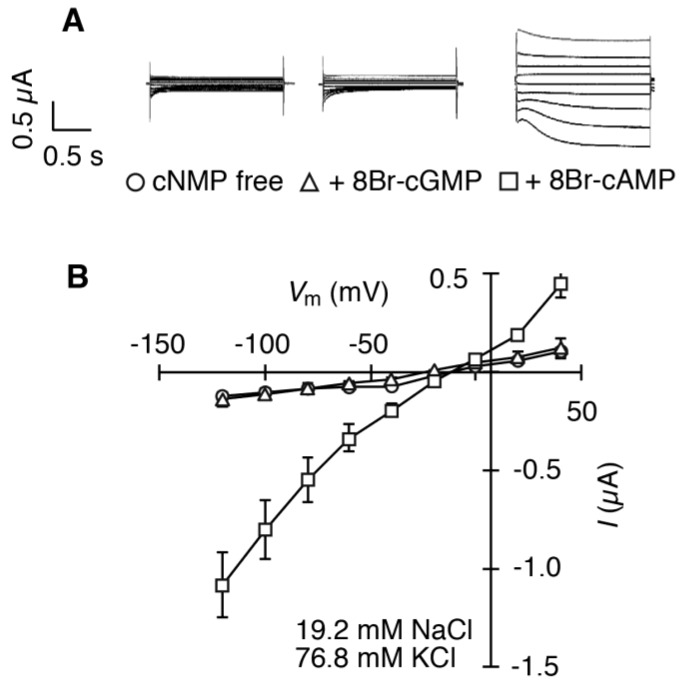
Current/voltage relationship of *HvCNGC2-3*-expressing oocytes in a 1:4 mixture of Na^+^ and K^+^ media. Currents were recorded by the two-electrode voltage-clamp technique in a bathing solution containing 19.2 mM NaCl and 76.8 mM KCl in the absence of a pre-incubation with cyclic nucleoside monophosphates (cNMP-free, circles), and in the presence of a pre-incubation with 10 µM 8Br-cGMP (triangles) or 8Br-cAMP (squares) for 30 min. (**A**) representative raw recording traces; (**B**) current/voltage curve. Error bars indicate standard deviation (*n* = 9, 13 and 6 for cNMP-free, cGMP and cAMP, respectively). *I*, membrane current. *V*m, membrane potential.

**Figure 6:**
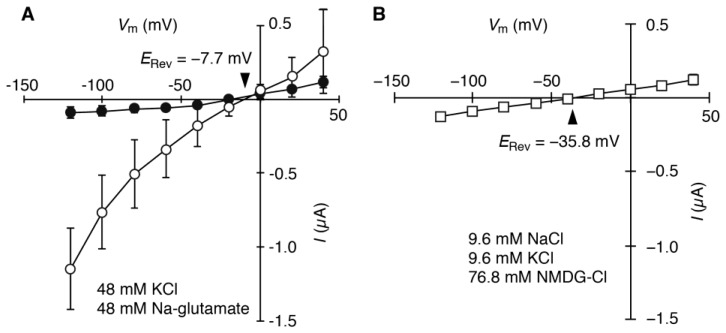
Current/voltage relationship of *HvCNGC2-3*-expressing oocytes in a gluconate-containing medium and *N*-methyl-d-glucamine-containing medium. Currents were analyzed with the two-electrode voltage-clamp technique. Oocytes were treated with 8Br-cAMP (10 μM) for 30 min before measurement. Oocytes expressing *HvCNGC2-3* (open symbols) or water-injected controls (closed symbols) were bathed in a solution containing 48 mM KCl and 48 mM Na-gluconate (**A**) and 9.6 mM KCl, 9.6 mM NaCl and 76.8 mM *N*-methyl-d-glucamine-Cl [NMCG-Cl] (**B**). Current values are means (*n* = 9 for water-injected control, *n* = 19 for *HvCNGC2-3* of gluconate-containing medium and *n* = 4 for *HvCNGC2-3* of *N*-methyl-d-glucamine-containing medium) ± SD. *E*_rev_, reversal potential.

To determine the charge-carrying ion of *HvCNGC2-3* cRNA-injected oocyte currents, reversal potentials were determined in different ion compositions. A slow-activated current was observed in a 1:4 mixture of Na^+^ and K^+^ media (Figure 5A,B), apparently indistinguishable from the current recorded in a 1:1 mixture (Figure 4A,B). This current was strongly activated by 8Br-cAMP and not observed in water-injected oocytes (−0.09 ± 0.04 µA at −100 mV) (Figure S2). The reversal potential of the 1:1 Na^+^/K^+^ mixture was determined as −8.7 ± 1.0 mV. In the 1:4 mixture, it was determined as −8.2 ± 0.5 mV. This strongly suggests that the permeability of HvCNGC2-3 to Na^+^ and K^+^ was almost equal. To test Cl^−^ permeability, we next determined the reversal potential in a bathing solution in which half the Cl^−^ was replaced with gluconate^−^ (48 mM Na^+^, 48 mM K^+^, 48 mM Cl^−^ and 48 mM gluconate^−^ in Figure 6A compared to 48 mM Na^+^, 48 mM K^+^ and 96 mM Cl^−^ in Figure 4). Essentially the same current was observed in *HvCNGC2-3* cRNA-injected oocytes (Figure 6A). The reversal potential examined in the 1:1 Cl^−^/gluconate^−^ solution was −7.7 ± 4.0 mV (*n* = 6). Additionally, the reversal potential determined in the medium containing 9.6 mM NaCl, 9.6 mM KCl and 76.8 mM *N*-methyl-d-glucamine-Cl was −36.8 ± 2.5 mV (*n* = 4) (Figure 6B), while the current was greatly reduced. This reduction of the current may be due to the suboptimal concentrations of Na^+^ and K^+^. No reversal potential shift in 1:1 Cl^−^/gluconate^−^ solution and the negative shift of the reversal potential in 1:1:8 Na^+^/K^+^/*N*-methyl-d-glucamine^+^ solution indicate that Cl^−^ is not the charge-carrying ion of the *HvCNGC2-3*-injected oocytes.

The permeability ratio of K^+^, Na^+^, and Cl^−^ (P_K_, P_Na_ and P_Cl_, respectively) was calculated as P_K_:P_Na_:P_Cl_ = 1:0.63:0.03, based on the Goldman–Hodgkin–Katz equation. This indicates that HvCNGC2-3 allows permeation of Na^+^ and K^+^ to a similar extent, but Cl^−^ permeation is virtually excluded.

cNMP-activated currents associated with HvCNGC2-3-injected oocyte was not observed in the bathing solution in which NaCl and KCl were replaced with CaCl_2_ (Y. Nobukiyo, unpublished data, in preparation), suggesting HvCNGC2-3 does not conduct Ca^2+^, while it is possible that we have not used appropriate condition to observe Ca^2+^ currents. 

### 2.3. Presence of Atypical Ion-Selective Motifs of HvCNGC2-3

**Figure 7:**
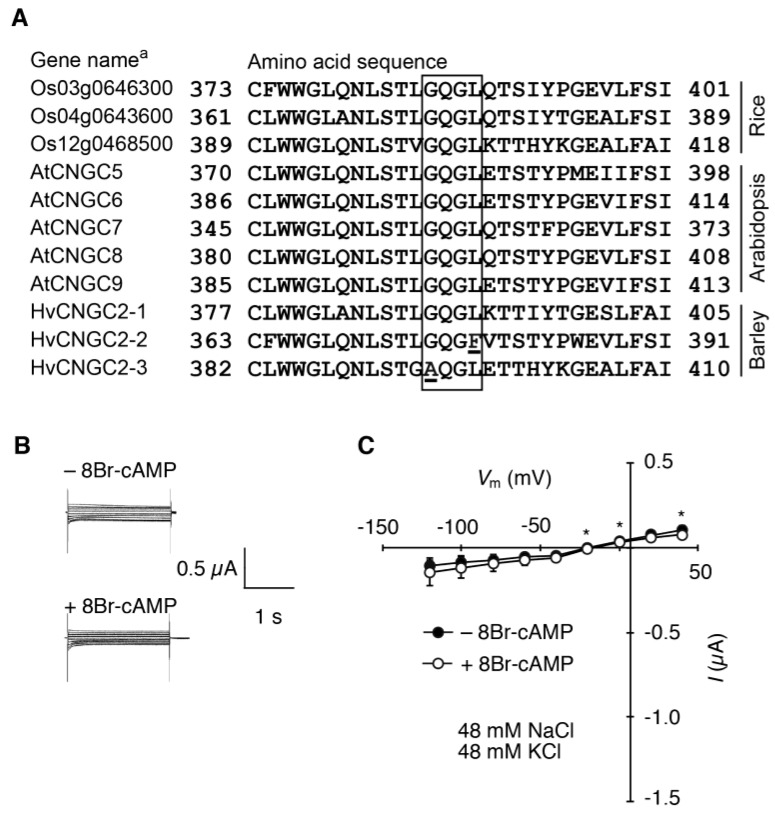
Alignment of deduced amino acid sequences of the putative ion-selective pore-forming motif (G/AQGL) of group-II CNGSs and voltage-clamp analysis of oocytes expressing *HvCNGC2-3A394G*. (**A**) the ion-selective motif is highlighted by box. Numbers indicate the amino acid positions. Alanine residue in the ion-selective motif of HvCNGC2-3 and Phenylalanine residue in the ion-selective motif of HvCNGC2-2 are underlined. ^a^ Gene names of rice are according to Rice Annotation Project identifiers (URL: https://rapdb.dna.affrc.go.jp). Arabidopsis Genome Initiative codes of Arabidopsis CNGCs are shown in Table S2. Gene names of barley CNGCs were allocated by this study (for accession number of each CNGC gene of barley, see Table S1); (**B**) representative raw current traces. Bathing solution contained 48 mM KCl and 48 mM NaCl; (**C**) current–voltage relationship. Oocytes were treated with cNMP-free bathing solution (closed symbols) or with 8Br-cAMP bathing solution (10 μM, open symbols) 30 min before measurements. Current values are means (*n* = 12 for cNMP-free and *n* = 10 for 8Br-cAMP) ± SD. Asterisks indicate a significant difference of the means between oocytes treated with and without 8Br-cAMP (Student’s *t*-test, *p* < 0.05).

Alignments of the deduced amino acid sequences of the putative pore-forming region of the group-II CNGCs [3] are shown in Figure 7A. The alignment of the CNGCs of *Arabidopsis,* rice, and barley demonstrated a well-conserved 4-amino acid motif. Nine sequences out of 11 have the consensus ion-selective motif GQGL. Unlike the others, HvCNGC2-3 has an alanine instead of a glycine amino acid and HvCNGC2-2 has a phenylalanine instead of a leucine amino acid in the motif. We hypothesized that this characteristic motif of HvCNGC2-3 results in unique ion selectivity. To test this hypothesis, we constructed *HvCNGC2-3A394G* that replaced alanine at position 394 with glycine, and characterized its electrophysiological properties by TEVC. The currents of *HvCNGC2-3A394G*-injected oocytes were examined in the 1:1 NaCl/KCl bath solution (Figure 7B,C). Critically, no current was observed both in the presence (−0.09 ± 0.04 µA at −100 mV) and absence (−0.12 ± 0.07 µA at −100 mV) of 8Br-cAMP, in stark contrast to wild-type HvCNGC2-3. This suggests that the atypical motif, AQGL, has a crucial role in the ion permeability or the activation mechanism of HvCNGC2-3.

## 3. Discussion

Electrophysiological characterizations of plant CNGCs have been demonstrated very poorly to date [14,15,16]. One of the reasons is the unsuccessful measurement of ion current in heterologous expression systems. This difficulty is likely due to unexpected ion selectivity or unknown activation mechanisms of plant CNGCs. Plant CNGCs have been postulated to function as non-selective cation channels [3,45]. Indeed, genetic evidence has demonstrated that Ca^2+^ is associated with phenotypes of mutants of *AtCNGC2*, *AtCNGC4*, *AtCNGC5*, *AtCNGC6*, *AtCNGC16* and *AtCNGC18* genes [25,29,42]. Electrophysiological evidence for their Ca^2+^ permeability has been shown in some CNGCs [18,19,20,21]. However, a plant CNGC is reported not to permeate Ca^2+^; AtCNGC2 was examined in the *X. laevis* oocyte heterologous expression system and shown to allow permeation of K^+^ instead of Ca^2+^ [14,15]. AtCNGC10 enables K^+^ permeation when examined in a heterologous expression system of HEK 293 cells [16]. We hypothesized that plant CNGCs may have unique ion selectivity distinct from any that we could have anticipated based on the similarity of amino acid sequences with animal CNGCs. In this study, we successfully observed the current of barley HvCNGC2-3 allowing permeation of K^+^ and Na^+^ in the co-presence of K^+^ and Na^+^ in the heterologous expression system. To our surprise, the ion current associated with HvCNGC2-3 was only observed when Na^+^ and K^+^ co-existed in the bath solution. This unique property has not been reported previously in any CNGC to the best of our knowledge. Permeability of HvCNGC2-3 to Na^+^ and K^+^ was almost equal, whereas that of Cl^−^ was substantially lower. The specific mechanism of activation of HvCNGC2-3 by the two ions remains unresolved. Nonetheless, this study provides novel insight into the ion selectivity and activation of plant CNGCs. Hyperpolarization-activated cyclic-nucleotide-gated (HCN) channels are animal ion channels of which gating is regulated by cyclic nucleotides and hyperpolarized membrane voltage [46]. HCN channels are permeable to Na^+^ and K^+^, being similar to HvCNGC2-3. Ion selective motif of human CNGCs is not strictly conserved (Table S3) that might be relevant for the low ion selectivity. On the other hand, the ion selective motif of human HCN channels is universally GYG. GYG motif is well established as K^+^ selective motif, but also able to conduct Na^+^ and Li^+^ as well [47]. Looking into the similarity of the motifs HCN channels and plant group II CNGC, there are two glycines sandwiching an amino acid residue with a relatively large size (Table S3). Whereas this structural similarity, we have no evidence that plant CNGC possessing the GQGL motif is involved in Na^+^ and/or K^+^ permeation. HCN channels conduct Li^+^ as well as other alkali ions [46], while HvCNGC2-3 did not conduct Li^+^ (Figure S3). Collectively, the AQGL motif of HvCNGC2-3 has unique ion selectivity among known cyclic nucleotide-gated channels.

Amino-acid sequences of the putative ion-selective pore-forming motif in plant CNGCs are unique to each group [3]. The CNGCs in group II generally possess a GQGL sequence at the corresponding position (Figure 7). However, HvCNGC2-3 possesses AQGL at that position. We examined whether this unique motif corresponds to the unique ion selectivity. As expected, point-mutated HvCNGC2-3, which has the general GQGL sequence instead of the unique AQGL, did not show a current in the presence of Na^+^ and K^+^. This indicates that the Na^+^-K^+^-activated Na^+^/K^+^ non-selective current of HvCNGC2-3 is unique and it is defined by the atypical ion-selective motif. This unique amino acid motif, which is located in the predicted ion-selective motif, may be involved in the unique ion selectivity or activation of the channel through unexplained mechanisms. This atypical motif is although not a solitary mutation in barley. AQGL motif is also found in Triticeae species, such as *Aegilops tauchii*, *Triticum urartu* and *T. aestivum*, but not in *Brachypodium distachyon*, *Zea mays* and *Sorgham bicolor* (Figure S4). The acquisition of this unique motif through evolution, conceivably a gain-of-function mutation, in a subset of group II CNGC of the common ancestor of barley and diploid wheat might have been beneficial for enabling Triticeae species to adapt to a certain environment. A unique motif of HvCNGC2-2 that is GQGF (Figure 7A), which is also found in *T. aestivum* (Figure S4) remains to be investigated.

Recently, it was reported that a double loss-of-function *Arabidopsis* mutant for *AtCNGC5* and *AtCNGC6*, which belong to group II, substantially lacked cGMP-activated Ca^2+^-permeable currents across the plasma membrane of guard cells [25]. The report showed that AtCNGC5- and AtCNGC6-associated currents were activated by cGMP, but not by cAMP. On the contrary, HvCNGC2-3 was not apparently activated by cGMP but activated by cAMP. This suggests that the specificity to cyclic nucleotides is variable even in the same group. Similar observations were found in mammals where the sensitivity of cyclic nucleotides was different among CNGCs. For example, an olfactory channel can be activated by physiological concentrations of both cAMP and cGMP [7,48], whereas photoreceptor channels are activated only by cGMP [4,8]. Cyclic nucleotide binding domain (CNBD) of AtCNGC5, AtCNGC6 and HvCNGC2-3 shares secondary structures with CNBD of the *Caenorhabditis elegans* CNGC, TAX4, and the human hyperpolarization-activated cyclic nucleotide-gated channel, HCN2 (Figure S5) according to the prediction by Jpred 4 Protein Secondary Structure Prediction Server [49]. Recent crystallography studies of CNBD suggested that four amino acid residues, the valine in β4, and the valine/methionine and the leucine in the β5 together with the lysine/arginine at the carboxyl-terminus interact with purine ring of cGMP [50,51]. We speculate that variation in these residues in β4 and β5 (Figure S5) is involved in the difference in cNMP specificity between these CNGCs. The physiological roles of cyclic nucleotide species in salinity stress and HvCNGC2-3 function in barley require further investigation. 

HvCNGC2-3 showed a weak voltage-dependent gating. Cyclic nucleotide-activated currents were generally activated by hyperpolarization [14,22,25], although non-voltage-dependent currents of a CNGC were also reported [16]. Apparent gating charge of HvCNGC2-3 was very low (0.016) to compare with other channels (for example, gating charge of KAT1 and KAT2 were 1.6 and 2.5, respectively [52]) and indicated a gradual voltage dependency. The channel remained partially opened with *P_min_* = 0.46. Therefore, it is conceivable that voltage-dependency of this CNGC channel is not physiologically meaningful. The τ of HvCNGC2-3 was determined as approximately 0.6 s. This is slower than the inward-rectifying K^+^ channel, KAT1 (0.1–0.2 s) [53]. The weak-rectifying property of HvCNGC2-3 is similar to the Arabidopsis K_weak_ channel, AKT2/3 [54]. Analogous to six membrane-spanning voltage-dependent K^+^ channels, the peripheral membrane spanning domains, S1 to S4, have voltage-sensing arginine or lysine residues [55]. In HvCNGC2-3, four arginine residues and a lysine are present (R150, R196, R243, R247 and K229) through S2 to S4. Some of these amino acid residues may be involved in the voltage sensing of HvCNGC2-3, as R197 in S4 domain of AKT2/3 is critical in weak rectification or leak-like channel gating [54]. The position of basic amino acids in the voltage-sensing S4 domain of animal CNGCs and HCN channels is apparently dissimilar to that of plants as shown in Table S3 [56]. 

Barley is a drought and salt stress resistant plant [57] and Na^+^ content in xylem sap of barley is maintained in the millimolar range even under salt stress (~200 mM NaCl) by compartmentalizing Na^+^ in the root cortex to reduce the xylem loading [58]. It is conceivable that the root-expressed HvCNGC2-3 functions in response to salinity stress by increasing the overall osmolality or sequestrating Na^+^ into vacuoles in roots. This unique gene might be inherited in the barley genome to withstand in the semi-arid climate where their ancestors emerged [59]. It is reported that in *Arabidopsis*, salt stress increases the cGMP level in the roots [60] and cGMP inhibits Na^+^-permeable VIC channels [24]. These results indicate that cGMP can mitigate Na^+^ entry to root cells in Arabidopsis. Unlike *Arabidopsis*, the Na^+^ and K^+^ permeating CNGCs of barley may play a role in balancing the ratio of Na^+^ and K^+^ in cells by the uptake of Na^+^ together with K^+^, and thus sustain osmotic potential in barley roots [58], so that the roots can take up water under low soil water potential. VIC is suggested to function as a Na^+^ importer in barley [1,2]. *HvCNGC2-3* may be a plausible candidate gene for Na^+^-permeable VIC in addition to wheat *LCT1* gene [61]. It should be also taken account of the function as a Na^+^ sensor that evoke an excitation of a non-excitatory cellular response in roots by K^+^ influx, which gives rise to membrane depolarization. 

## 4. Materials and Methods

### 4.1. Plant Materials, cDNA Isolation and Expression of HvCNGC2-3 in Xenopus laevis Oocytes

Seedlings of barley (*Hordeum vulgare* cv. Haruna-nijyo) were hydroponically grown in the nutrient solution in a temperature-controlled room at 25 ± 2 °C with aeration as described previously [62]. The seeds were originally provided from Barley and Wild Plant Resource Center, Okayama University and annually amplified in the field of Institute of Plant Science and Resources, Okayama University. The nutrient solution contained 4 mM KNO_3_, 1 mM NaH_2_PO_4_, 1 mM MgSO_4_, 1 mM CaCl_2_ and 1 mg L^−1^ iron(III)-citrate. The pH was adjusted to 5.5 with NaOH. The light cycle was 14-h light/10-h dark. Light intensity was 150 μmol photons m^−2^ s^−1^. Plantlux lamps (Toshiba FL20T8BR/18, Yokosuka, Japan) were used for illumination. 

Total RNA was isolated using the RNeasy Plant Mini Kit (Qiagen, Hilden, Germany) from whole roots of 5-day-old seedlings. After reverse-transcription with moloney murine leukemia virus reverse transcriptase (Invitrogen Life Technologies Japan, Osaka, Japan), full-length *HvCNGC2-3* cDNA was amplified using KOD-FX DNA polymerase (Toyobo Life Science, Osaka, Japan) with a pair of primers, FW: TGCAGCAGCATGCAGTCGCCTCCGCCCTAG, RV: CGCGTCAGATATGTGTGGCACCCCGCAGTTG. The coding region of *HvCNGC2-3* was subcloned into a pXβG-ev1 plasmid [62].

Female *X. laevis* were procured from either of the two companies (Watanabe Zoushoku, Hyogo, Japan and Hamamatsu Seibutsu Kyozai Ltd., Hamamatsu, Japan) and cared for in the laboratory. Oocytes were harvested as described previously [62]. Synthesis and microinjection (50 ng per oocyte) of *HvCNGC2-3* complementary RNA (cRNA) into oocytes were performed as described previously [63]. The injected oocytes were incubated at 18 °C for 24–48 h in modified Barth’s solution (MBS) [64] until electrophysiological experiments were performed. The experiments using frog oocytes were approved by the Animal Care and Use Committee, Okayama University (approval number OKU-2017271) that follows the related international and domestic regulations.

### 4.2. Phylogenetic Analysis

The phylogenetic tree was constructed by the neighbor-joining method using CLASTAL W [65,66] from rice, *Arabidopsis* and barley full-length CNGC coding sequences.

### 4.3. Analysis of the Expression of HvCNGC2-3 mRNA in Barley Plants

Seedlings were grown as mentioned above. When the second leaf emerged, plants were dissected into 5 parts (leaf blade, mature root (above 2 cm from the tip), root tip (within 2 cm from the tip), coleoptile and basal nodes) with a scalpel and snap-frozen in liquid nitrogen to isolate RNA using the Sepasol-RNAI super G solution (Nacalai Tesque, Kyoto, Japan) according to the manufacturer’s instruction. Single strand cDNA was synthesized using ReverTra Ace^®^ qPCR RT Master Mix with gDNA Remover kit (FSQ-301S, Toyobo Co. Ltd., Osaka, Japan). cDNA fragments of *HvCNGC2-3* were amplified with a set of primers, HvCNGC2-3FW: CGTCGACGAGATGTTCTTCA and HvCNGC2-3RV: ACCTTGACACCAGGAACGTC. A primer set of Elongation factor 1α (*EF1α*) was used for the internal control, HvEF1αFW: ATGGGCAAGGAAAAGATCCA and HvEF1αRV: TTCCAATGCCACCAATCTTG. Polymerase chain reactions (PCR) were performed in a thermal cycler (Bio-Rad Laboratories, Inc., Hercules, CA, USA) starting with polymerase activation at 95 °C for 2 min and 24 cycles of 95 °C for 15 s, 60 °C for 15 s and 72 °C for 60 s followed by a final extension at 72 °C for 10 min for *HvEF1α*. PCR for *HvCNGC2-3* started with polymerase activation at 95 °C for 2 min and 36 cycles of 95 °C for 15 s, 60 °C for 15 s and 72 °C for 60 s followed by a final extension at 72 °C for 10 min. In both cases, Takara Ex Taq HS polymerase (Takara Bio Inc., Kusatsu, Japan) was used. PCR was performed in triplicate and the amplified products were subjected to an agarose gel electrophoresis (1% for *HvEF1α* and 2% for *HvCNGC2-3*) and visualized with ethidium bromide staining (0.5 µg mL^−1^). DNA size marker in Figure 2 was 1 kb DNA ladder (New England Biolabs, Ipswich, MA, USA).

### 4.4. Electrophysiology

Oocytes injected with water or *HvCNGC2-3* cRNA were incubated in MBS supplemented with 10 μM (final concentration) 8-bromoadenosine cyclic monophosphate (8Br-cAMP, Sigma B5386, St Louis, MO, USA), 10 µM 8-bromoguanosine cyclic monophosphate (8Br-cGMP, Sigma B1381) or without supplementation of a nucleoside 3′,5′-cyclic monophosphate (cNMP) for 30 min prior to the two-electrode voltage-clamp (TEVC) measurement. TEVC recordings were performed with a two-glass-electrode voltage amplifier (Axoclamp 900A Microelectrode Amplifier; Molecular Devices, Sunnyvale, CA, USA) in a Na^+^ medium (96 mM NaCl, 1.8 mM CaCl_2_, 1.8 mM MgCl_2_ and 10 mM HEPES, pH7.5 with NaOH), or K^+^ medium (96 mM KCl and all else identical to the solution above except that KOH was used instead of NaOH), or a mixture of Na^+^ and K^+^ media with any given ratio (see text). Data were acquired using Clampex software (version 10.3, Molecular Devices, San Jose, CA, USA). Osmotic potential of the solutions (200 mmol/kg) was checked routinely with a vapor pressure osmometer (Wescor 5200, Wescor Inc., Logan, UT, USA). Voltage steps (2 s each) were applied from 40 mV to −120 mV in 20-mV decrements. The holding potential was −30 mV. All procedures were carried out at 20 ± 2 °C. The half-activation time of membrane current (τ) activated by a voltage shift from −30 mV to −120 mV was calculated by fitting to the logarithmic function ($I_{t}=I_{t=0}\cdot e^{-t/\tau }+I_{t=\infty }$) with the least square regression, where *I* indicates membrane current, and *e* and *t* are Napier’s constant and time, respectively. Current amplitude, applied voltage and τ were analyzed with Clampfit software (version 10.3, Molecular Devices). Apparent gating charge and half-activation membrane potential were determined by non-linear regression least squares fitting to the modified Boltzmann equation exhibiting a voltage-independent minimum open probability (*P_min_*) and a voltage-dependent term:

(1) $$P_{\left(V\right)}=\left(P_{max}-P_{min}\right)/\left(1+e^{z\delta F\left(V-V_{0.5}\right)/RT}\right)+P_{min},$$

with *zδ* being the apparent gating charge where *z* is the charge on the particle moving and *δ* is the fraction of the membrane field through which the charge moves, *P_max_* is the apparent maximum open probability and *V*_0.5_ the half-activation membrane potential based on relative open probability (*P*_o_) normalized to the maximum conductance [67]. Reversal potential was estimated from the *x*-intercept by the linear line connecting two data of membrane currents closest to the *x*-axis at both sides.

### 4.5. Amino Acid Substitution

The amino acid replacement of HvCNGC2-3 was carried out according to [68], using a pair of primers: CCTCAGCACCGGCCAACAGGGGCTAGAGAC and GTCTCTAGCCCCTGTTGGCCGGTGCTGAGG. The full length DNA sequence of *HvCNGC2-3* open reading frame, which has A394G mutation cloned in the pXβG-ev1 plasmid was confirmed by Sanger’s sequencing.

### 4.6. Statistics

Difference between the means of datasets was examined by two-tailed Student’s *t*-test or analysis of variance followed by Dunnett’s multiple comparisons test as indicated in figure legends.

## 5. Conclusions

Our electrophysiological analysis of the CNGC of barley, HvCNGC2-3, is the first to demonstrate that a CNGC is activated by the co-presence of Na^+^ and K^+^. RT-PCR experiments localized the expression of HvCNGC2-3 mRNA mainly in mature roots. These findings suggest a physiological role for HvCNGC2-3, wherein it functions to transport alkali-metal cations into roots during salinity stress. The amino-acid sequence of the putative ion-selective motif of HvCNGC2-3 was not typical compared with other plant CNGCs in group II. The substitution of the atypical amino acid residue with the canonical residue caused a loss of ion permeability. Thus, the electrophysiological properties of HvCNGC2-3 may be unique among group-II CNGCs.

## Supplementary Materials

The following are available online at http://www.mdpi.com/2223-7747/7/3/61/s1, Table S1: Accession number of nucleotide sequence and peptide sequence of barley CNGCs, Table S2: Gene names of Arabidopsis CNGCs and their Arabidopsis Genome Initiative (AGI) code, Table S3: Comparison of deduced ion selective motifs and S4 domains in CNG and HCN channels in human and group II CNGC in barley, Figure S1: Current/voltage relationship of HvCNGC2-3 expressing oocytes in the presence of 8Br-cGMP, Figure S2: Current/voltage relationship of water-injected oocytes, Figure S3: Effects of substitution of Na^+^ and K^+^ on the current of *HvCNGC2-3* cRNA-injected oocyte, Figure S4: Putative ion-selective pore-forming motifs of representative group II CNGCs of glasses, Figure S5: Secondary structure prediction of the cyclic nucleotide binding domains.
