# Supplementary material for: A Cyclic Nucleotide-Gated Channel, HvCNGC2-3, Is Activated by the Co-Presence of Na+ and K+ and Permeable to Na+ and K+ Non-Selectively
Source: Plants (Basel). 2018 Jul 26;7(3):61. doi: 10.3390/plants7030061 (PMC6161278; doi:10.3390/plants7030061)
Supplement: Supplementary file 1 [file plants-07-00061-s001.pdf]

A Cyclic Nucleotide-Gated Channel, HvCNGC2-3, is Activated by the Co-presence of Na^+ and K^+ and Permeates Na^+ and K^+ Non-selectively

Izumi C. Mori, Yuichi Nobukiyo, Yoshiki Nakahara, Mineo Shibasaka, Takuya Furuichi and Maki Katsuhara

Supplementary Materials: Table S1: Accession number of nucleotide sequence and peptide sequence of barley CNGCs, Table S2: Gene name of Arabidopsis CNGCs and their Arabidopsis Genome Initiative (AGI) code, Table S3: Comparison of deduced ion selective motifs and S4 domains in CNG and HCN channels in human and group 2 CNGC in barley, Figure S1: Current/voltage relationship of HvCNGC2-3 expressing oocytes in the presence of 8Br-cGMP, Figure S2: Current/voltage relationship of water-injected oocytes, Figure S3: Effects of substitution of Na^+ and K^+ on the current of *HvCNGC2-3* cRNA-injected oocyte, Figure S4: Putative ion-selective pore-forming motifs of representative subgroup II CNGCs of glasses, Figure S5: Secondary structure prediction of the cyclic nucleotide binding domains.

Table S1. Accession number of nucleotide sequence and protein sequence of barley CNGCs

Gene name	Accession number	
	Nucleotide sequence	Protein sequence
HvCNGC1-1	AK366449.1	BAJ97652.1
HvCNGC2-1	AK373237.1	BAK04434.1
HvCNGC2-2	AK370933.1	BAK02131.1
HvCNGC2-3	AK376248.1	BAK07443.1
HvCNGC3-1	AK376248.1	BAK07912.1
HvCNGC3-2	AK355803.1	BAJ86607.1
HvCNGC3-3	AK371848.1	BAK03046.1
HvCNGC4a-1	AK365205.1	BAJ96408.1
HvCNGC4b-1	AK367927.1	BAJ99130.1

Table S2. Gene name of Arabidopsis CNGCs and their Arabidopsis Genome Initiative (AGI) code

Gene name	AGI code
AtCNGC1	At5g53130
AtCNGC2	At5g15410
AtCNGC3	At2g46430
AtCNGC4	At5g54250
AtCNGC5	At5g57940
AtCNGC6	At2g23980
AtCNGC7	At1g15990
AtCNGC8	At1g19780
AtCNGC9	At4g30560
AtCNGC10	At1g01340
AtCNGC11	At2g46440
AtCNGC12	At2g46450
AtCNGC13	At4g01010
AtCNGC14	At2g24610
AtCNGC15	At2g28260
AtCNGC16	At3g48010
AtCNGC17	At4g30360
AtCNGC18	At5g14870
AtCNGC19	At3g17690
AtCNGC20	At3g17700

The data was acquired from The Arabidopsis Information Resource (http://www.arabidopsis.org).

Table S3. Comparison of deduced ion selective motifs and S4 domains in CNG and HCN channels in human and group II CNGC in barley

Gene	Ion selective motif	S4 domain
Human CNG channels (non-selective, voltage-independent)		
CNGA1	GGL	RLNRLLRFSRMFEFFQR
CNGA2	GGL	RFNRLLHFARMFEFFDR
CNGA3	GET	RFNRLLKFSRLFEFFDR
CNGA4	GET	RLNRFLRAPRLFEAFDR
CNGB1	GET	RLPRCLKYMAFFEFNSR
CNGB3	GDT	RANRMLKYTSFFEFNHH
Human HCN channels (K^+ and Na^+-permeable, voltage-dependent)		
HCN1*	GYG	RALRIVRFTKILSLLRLLRLSRLIR
Barley group 2 CNGCs (K^+ and Na^+-permeable, partially voltage-dependent)		
HvCNGC2-1	GQGL	KDRLLSIIIAQYVPRLVRIYPLS
HvCNGC2-2	GQGF	KDALVWVVLCQYIPRLLRIFPVT
HvCNGC2-3	AQGL	KTALFFIVLTQYLPRLVRFYPII

Magenta and green indicate basic and acidic amino acid residues, respectively. Accession numbers of human genes are as follow: CNGA1, NP_001136036.1; CNGA2, EAW99409.1; CNGA3, NP_001289.1; CNGA4, XP_016872706.1; CNGB1, XP_011521172.1; CNGB3, XP_011515440.1; and HCN1, NP_066550.2. Accession numbers of barley genes are shown in Table S1. *Amino acid sequences of ion selective motif and S4 domain in HCN2 (NP_001185.3), HCN3 (NP_065948.1) and HCN4 (NP_005468.1) are identical to HCN1.

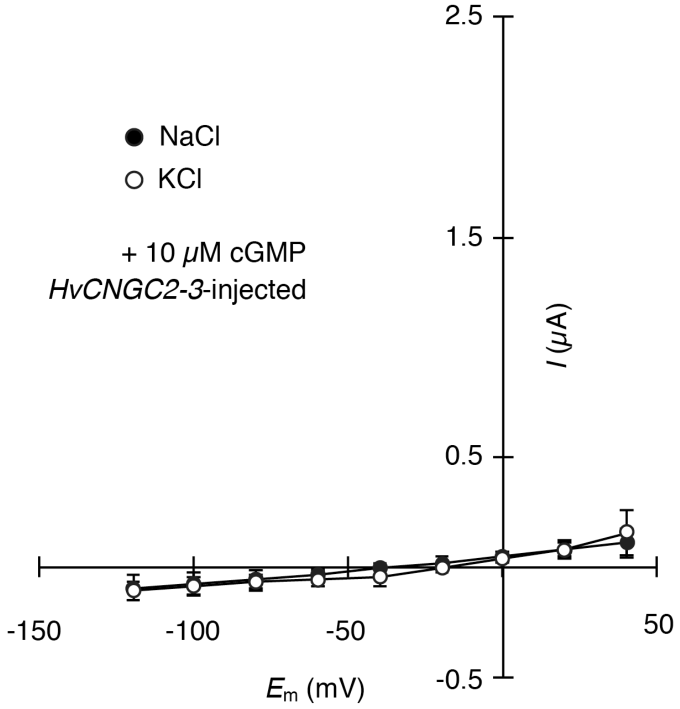

Figure S1. Current/voltage relationship of HvCNGC2-3 expressing oocytes in the presence of 8Br-cGMP. Currents were recorded by the two-electrode voltage-clamp. Bath solution contained 96 mM NaCl (closed symbols) or 96 mM KCl (open symbols). Oocytes were treated with 10 μM 8Br-cGMP 30 min before measurements. Current values are means (n=15 [NaCl] and 10 [KCl]) ± SD. *I*, membrane current. E_m, membrane potential.

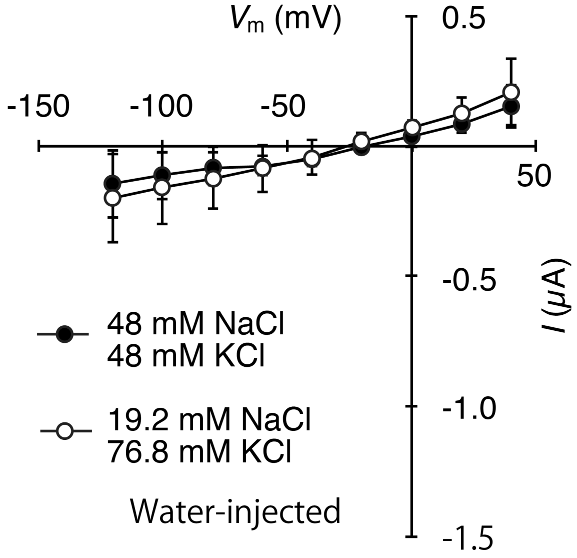

Figure S2. Current/voltage relationship of water-injected oocytes. Currents were recorded by the two-electrode voltage-clamp in bath solution containing 19.2 mM NaCl and 76.8 mM KCl (open symbols, n = 10), and 48 mM NaCl and 48 mM KCl (closed symbols, n = 11) in the presence of 10 μM 8Br-cAMP.

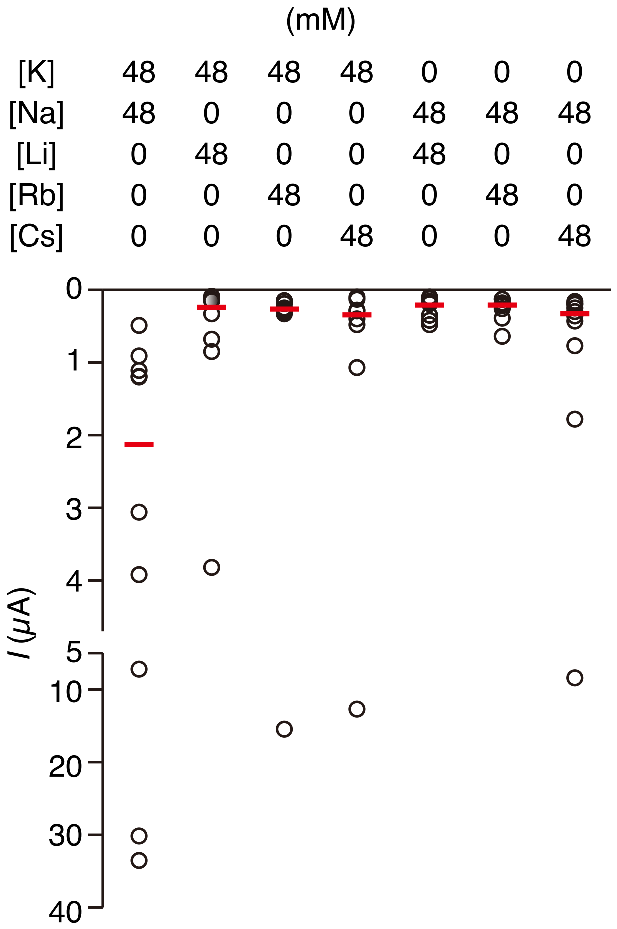

Figure S3. Effects of substitution of Na^+ and K^+ on the current of *HvCNGC2-3* cRNA-injected oocyte. Currents at –120 mV was plotted. Open circles indicate each measurement (n=10). Red lines indicate the median. Either of Na^+ or K^+ was substituted with Li^+, Rb^+ or Cs^+. Mann-Whitney *u* test suggested that significant difference of the mean of current amplitude was observed in HvCNGC2-3-injected oocytes from water-injected control only in 48 mM K^+ and 48 mM Na^+ bathing solution ($\alpha = 0.05$).

```
Triticeae
  Hordeum vulgare (Barley)
   HvCNGC2-1                              CLWWGLANLSTLGQGLK-TTIYTGESLF
   HvCNGC2-2                              CFWWGLQNLSTLGQGFV-TSTYPWEVLF
   HvCNGC2-3                              CLWWGLQNLSTGAQGLE-TTHYKGEALF

  Aegilops tauschii (Tausch's goatgrass)
   XP_020165922.1                         CLWWGLANLSTLGQGLK-TTIYTGESLF
   EMT29601.1                             CLWWGLQNLSTGAQGLE-TTHYKGEALF
   EMT29672.1                             CLWWGLQNLSSGAQGLE-TTHYKGEALF

  Triticum urartu (Red wild einkorn)
   T1NH13_TRIUA                           CLWWGLQNLSTGAQGLE-TTHYKGEALF

  Triticum aestivum (Common wheat)
   TRIAE_CS42_2AL_TGACv1_097035_AA0322740.1   CFWWGLQNLSTLGQGFV-TSTYPWEVLF
   TRIAE_CS42_2BL_TGACv1_130157_AA0405260.1   CFWWGLQNLSTLGQGFV-TSTYPWEVLF
   TRIAE_CS42_2DL_TGACv1_160734_AA0553580.1   CFWWGLQNLSTLGQGFV-TSTYPWEVLF
   TRIAE_CS42_1AS_TGACv1_019890_AA0072520.1   CLWWGLQNLSTGAQGLE-TTHYKGEALF
   TRIAE_CS42_1BS_TGACv1_049361_AA0150610.1   CLWWGLQNLSTGAQGLE-TTHYKGEALF
   TRIAE_CS42_1DS_TGACv1_080801_AA0253910.1   CLWWGLQNLSTGAQGLE-TTHYKGEALF
   TRIAE_CS42_5AL_TGACv1_375657_AA1225250.1   CLWWGLQNLSSGAQGLE-TTHYKGEALF
   TRIAE_CS42_5BL_TGACv1_405743_AA1334310.1   CLWWGLQNLSSGAQGLE-TTHYKGEALF
   TRIAE_CS42_5DL_TGACv1_437676_AA1467100.1   CLWWGLQNLSSGAQGLE-TTHYKGEALF

Brachypodieae
  Brachypodium distachyon (Purple false brome)
   Bradi5g23700.1                         CFWWGLQNLSTLGQGFT-TSTYPGEVLF
   Bradi1g13740.1                         CLWWGLANLSTLGQGLK-TTIYTGEALF
   Bradi1g78010.1                         CLWWGLQNLSTVGQGLDQTTHYKGEALF

Andropogoneae
  Zea mays (Maize)
   GRMZM2G023037                          CFWWGLQNLSTLGQGLL-TSTYPGEVLF
   AC197150.3                             CLWWGLQNLSTIGQGLE-TTHYKGEQLF

  Sorgham bicolor (Sorgham)
   Sb01g013500.1                          CLWWGLANLSTLGQGLK-TSIYTGEALF
   Sb06g030420.1                          CFWWGLQNLSTLGQGLM-TSTYTGEVLF
   Sb08g021830.1                          CLWWGLQNLSTIGQGLE-TTHYKGEQLF

Oryzeae
  Oryza sativa (Rice)
   Os03g0646300                           CLWWGLANLSTLGQGLQ-TSIYTGEALF
   Os04g0643600                           CFWWGLQNLSTLGQGLQ-TSIYPGEVLF
   Os12g0468500                           CLWWGLQNLSTVGQGLK-TTHYKGEALF
```

Figure S4. Putative ion-selective pore-forming motifs of representative subgroup II CNGCs of glasses. Twenty seven CNGCs showing >60% identity with HvCNGC2-3 was BLAST searched in two databases, EnsemblePlants (http://plants.ensembl.org) for *A. tauschii*, *T. urartu* and *T. aestivum* and PlantGDB (http://www.plantgdb.org) for *B. distachyon*, *Z. mays*, *S. bicolor* and *O. sativa*. Amino acid sequence of the putative ion-selective motif and their short flanking region were aligned manually. Glycine and alanine residues in the motif were highlighted blue and red, respectively. Gene names are as designated in each database.

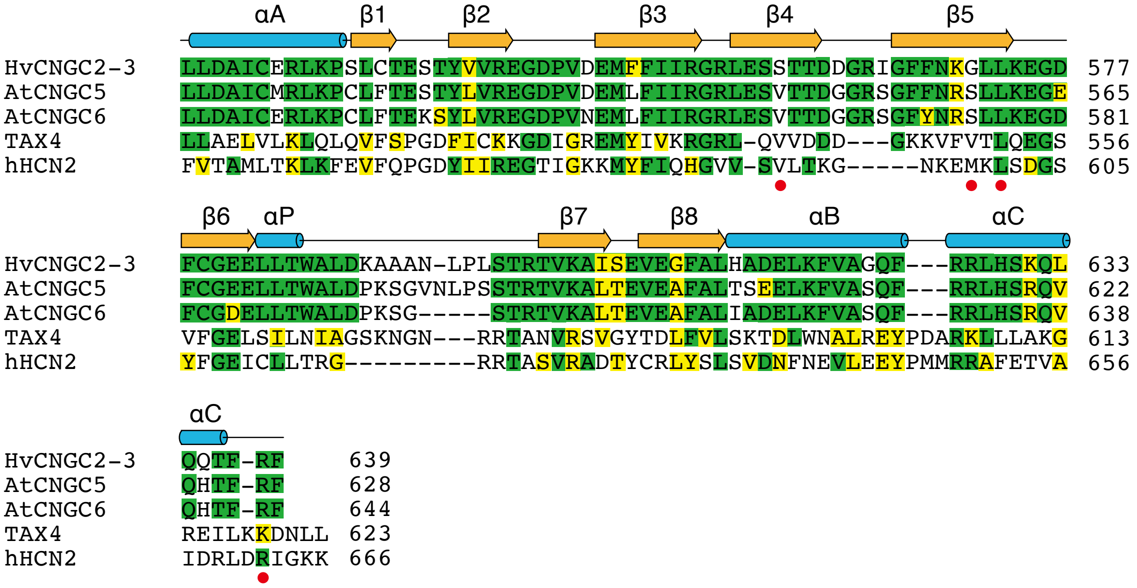

Figure S5. Secondary structure prediction of the cyclic nucleotide binding domains. Green and yellow boxes indicate identical and similar amino acid residues, respectively. Cyan cylinders and orange arrows indicate predicted α-helix and β-sheet structures. Red dots indicate amino acids responsible for interaction with cyclic nucleotides (ref [43, 44]).
